# Transcriptomic, biochemical and physio-anatomical investigations shed more light on responses to drought stress in two contrasting sesame genotypes

**DOI:** 10.1038/s41598-017-09397-6

**Published:** 2017-08-18

**Authors:** Komivi Dossa, Donghua Li, Linhai Wang, Xiaomin Zheng, Aili Liu, Jingyin Yu, Xin Wei, Rong Zhou, Daniel Fonceka, Diaga Diouf, Boshou Liao, Ndiaga Cissé, Xiurong Zhang

**Affiliations:** 1 Oil Crops Research Institute of the Chinese Academy of Agricultural Sciences, Key Laboratory of Biology and Genetic Improvement of Oil Crops, Ministry of Agriculture, No. 2 Xudong 2nd Road, 430062 Wuhan, Hubei China; 2 Centre d’Etudes Régional pour l’Amélioration de l’Adaptation à la Sécheresse (CERAAS), BP 3320 Route de Khombole, Thiès, Senegal; 3 Laboratoire Campus de Biotechnologies Végétales, Département de Biologie Végétale, Faculté des Sciences et Techniques, Université Cheikh Anta Diop, BP 5005 Dakar-Fann, Code postal, 107000 Dakar, Senegal; 4 BGI-Shenzhen, Shenzhen, China; 5 Centre de coopération internationale en recherche agronomique pour le développement (CIRAD), UMR AGAP, F-34398 Montpellier, France

## Abstract

Sesame is an important oilseed crop with a high oil quality. It is prone to drought stress in the arid and semi-arid areas where it is widely grown. This study aims to decipher the response of tolerant (DT) and sensitive (DS) genotypes to progressive drought based on transcriptome, biochemical and physio-anatomical characterizations. Results indicated that under severe stress, DT relied on a well-functioning taproot while DS displayed a disintegrated root due to collapsed cortical cells. This was attributed to a higher accumulation of osmoprotectants and strong activity of antioxidant enzymes especially peroxidases in DT. From roots, DT could supply water to the aboveground tissues to ensure photosynthetic activities and improve endurance under stress. Temporal transcriptome sequencing under drought further confirmed that DT strongly activated genes related to antioxidant activity, osmoprotection and hormonal signaling pathways including abscisic acid and Ethylene. Furthermore, DT displayed unique differentially expressed genes in root functioning as peroxidases, interleukin receptor-associated kinase, heat shock proteins, APETALA2/ethylene-responsive element-binding protein and mitogen activated protein kinase, to effectively scavenge reactive oxygen species and preserve root cell integrity. Finally, 61 candidate genes conferring higher drought tolerance in DT were discovered and may constitute useful resources for drought tolerance improvement in sesame.

## Introduction

Drought is the primary abiotic constraint limiting crop production worldwide, owing to climate change and shortages of freshwater associated with population growth^1^. It seriously reduces the growth, productivity and quality of crop plants^2^. The increasing vulnerability to drought requires that resilient varieties capable of surviving drought conditions while maintaining good yield are developed^3^. Therefore, the understanding of plant resistance to drought is of paramount importance to provide insights into the resistance mechanisms against this abiotic stress at biochemical, physiological, and molecular levels. It is well documented that numerous anatomical, physiological and molecular components of plant performance, including root parameters, leaf features, osmoprotection system, reactive oxygen species (ROS) scavenging system, membrane stability and photosynthetic capacity, influence plant responses to drought^4^.

Plant–water relations are largely determined by several physiological characteristics including relative water content (RWC) of the leaves wherein drought stressed plants have lower RWC as compared to non-stressed ones^5^. Stomata closure represents the first barrier plants employ to avoid dehydration, with the trade-off of a reduced CO_2_ supply to the mesophyll^6^. This leads to the accumulation of ROS including superoxide (O_2_^−^), hydrogen peroxide (H_2_O_2_), hydroxyl radical (HO^−^) and singlet oxygen (^1^O_2_) mostly in the chloroplast and mitochondria causing oxidative damages to lipids, proteins, nucleic acids and the photosynthetic apparatus^7^. Scavenging of ROS by enzymatic and non-enzymatic systems, cell membrane stability, expression of aquaporins and stress proteins are vital mechanisms of drought tolerance. To detoxify ROS, plants can intrinsically develop different types of antioxidants including superoxide dismutase (SOD), peroxidases (POD) and catalase (CAT), Ascorbate peroxidase (APX) to reduce oxidative damage, conferring drought tolerance^2^. A number of studies have indicated that higher activity levels of antioxidant enzymes may contribute to better drought tolerance in plants^8^. In addition, the molecule malondialdehyde (MDA) has been associated with lipid peroxidation via an increased generation of ROS, and thus its quantification has been suggested as a general indicator for drought tolerance^9^.

At the molecular level, drought induces a cascade of changes in expression of both regulatory and functional genes^10^. Two important pathways of transcriptional networks under drought stress have been highlighted including an abscisic acid (ABA)-dependent signaling pathway and an ABA-independent regulatory network. The mechanisms of signal perception and transduction are the subject of extensive research and some important breakthroughs have been reviewed by Yoshida *et al*.^11^. Studying transcriptome dynamics can provide important insights into the functional elements of the genome, their expression patterns, and the regulation of transcribed regions in drought stress conditions. Impact of drought on gene expression has been intensely analysed in numerous species such as Arabidopsis^12^, rice^13^, maize^14^ and tomato^15^ by high-throughput transcriptomics.

Sesame (*Sesamum indicum* L.) is becoming an emerging oilseed crop in the world owing to its high oil yield (~55%), quality and stability^16^. It is mostly grown under rain fed conditions in arid and semi-arid areas where it is prone to terminal and intermittent droughts^17^. According to Sun *et al*.^18^, sesame is highly sensitive to drought stress during anthesis with devastating effects on the number of capsules per plant, grain yield as well as oil yield and quality. Vegetable oil consumption is expected to reach almost 200 billion kilograms by 2030, which will increase the demand for oil-rich crops^19^. Sesame has the potential to greatly contribute in meeting this demand but the cultivation of varieties that tolerate drought is a necessity^16, 20, 21^. Unfortunately, the mechanism of sesame responses to drought stress is still poorly understood^22–24^. In addition, to date, knowledge concerning transcriptomic response from drought-stressed sesame remains inexistent^25^. With the completion of the *de novo* assembly of the sesame genome^26^, it is now possible to profile gene expression in response to drought by sequencing. In this study, drought response mechanisms in sesame have been comprehensively explored through comparative RNA-seq between drought tolerant and sensitive genotypes along with biochemical and physio-anatomical dissections.

## Results

### Changes in root anatomy in response to water stress

**Figure 1:**
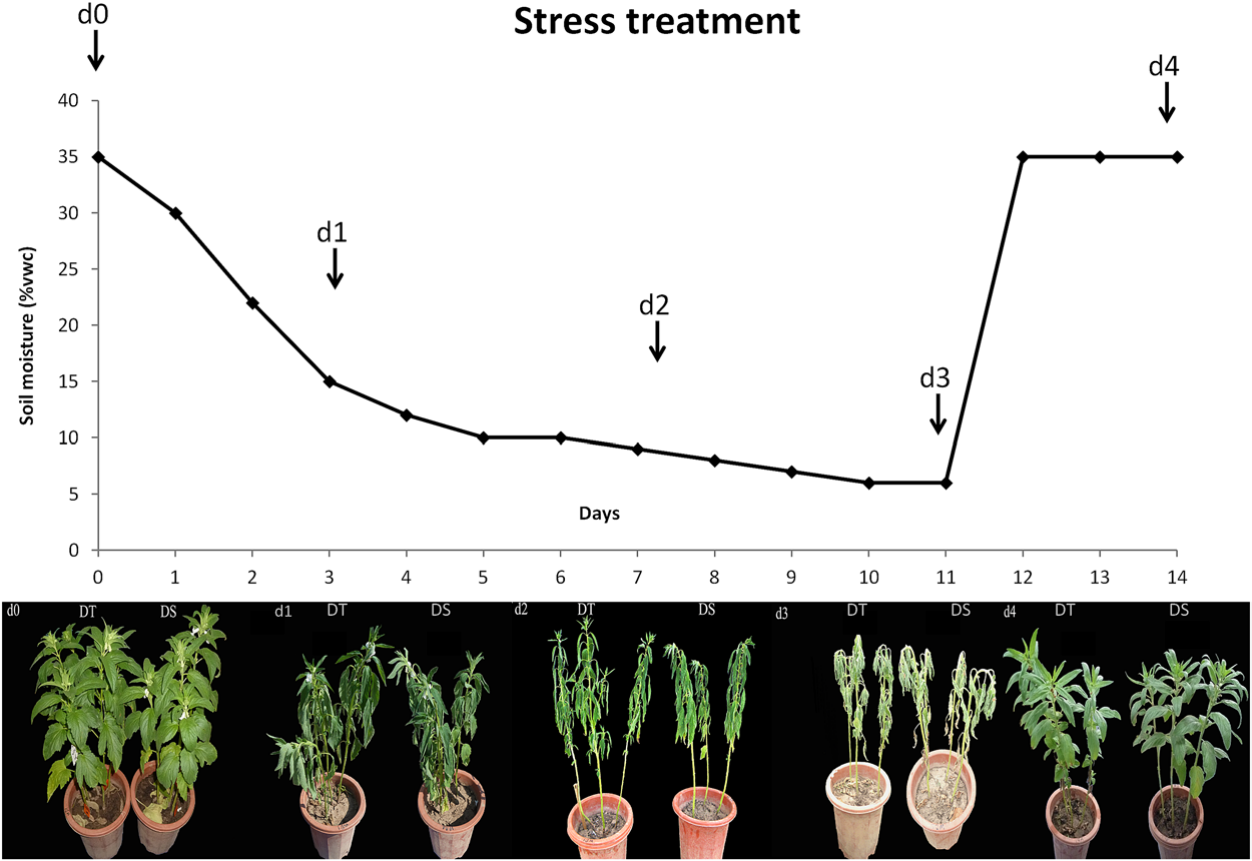
Morphological responses of sensitive (DS) and tolerant (DT) sesame genotypes. The leaves of DS suffer more damage than those of DT during stress. d_0_, d_1_, d_2_, d_3_ and d_4_: plants stressed for 0, 3, 7, 11 days and 3 days of recovery after rewatering, respectively.

**Figure 2:**
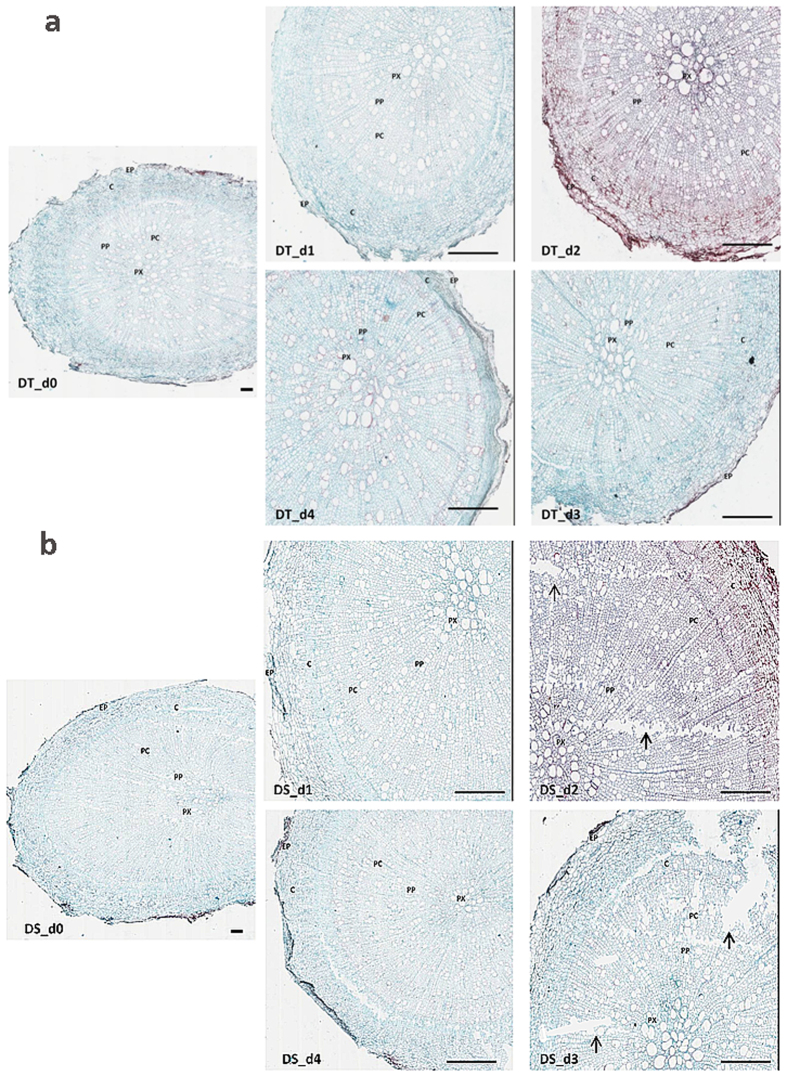
Transverse root sections of (**a**) tolerant (DT) and (**b**) sensitive (DS) sesame genotypes. d_0_, d_1_, d_2_, d_3_ and d_4_: root samples from plants stressed for 0, 3, 7, 11 days and 3 days of recovery after rewatering, respectively. Arrows in black indicate damaged cortex. EP: Epidermis; C: Cortex; PC: Parenchyma Cell; PP: Primary Phloem; PX: Primary Xylem. Scale bars = 100 µm.

Drought tolerant and sensitive sesame genotypes were submitted to gradual water deficit that led to slight, mild and severe wilting of the plants according to drought severity (Fig. 1). During the whole experiment, 40% of the sensitive genotype (DS) died whereas, 96% of the tolerant genotype (DT) could sustain the stress and recovered sharply after rewatering. Root samples were harvested at different time points and analysed to unravel drought impact on the organ anatomy. Prior to drought stress (d_0_), both genotypes presented a well-organised structure of their roots characterized by the presence of epidermic cells, followed by multiple layers of cortex tissue with several parenchymatous cells (Fig. 2a,b). Under drought stress, DT maintained a good organisation of the inner root structure even if very few intercellular spaces resulting from the collapse of the parenchyma cells could be observed (Fig. 2a). In contrast, at 7 days of stress (d_2_), DS exhibited a huge loss of cortical cells leading to a distortion of the organ morphology and the disintegration of the internal structure of the root (Fig. 2b). After rewatering, both genotypes displayed a good organisation of root structure suggesting a recovery from drought stress damages (Fig. 2a,b).

### Biochemical and physiological responses to water stress

**Figure 3:**
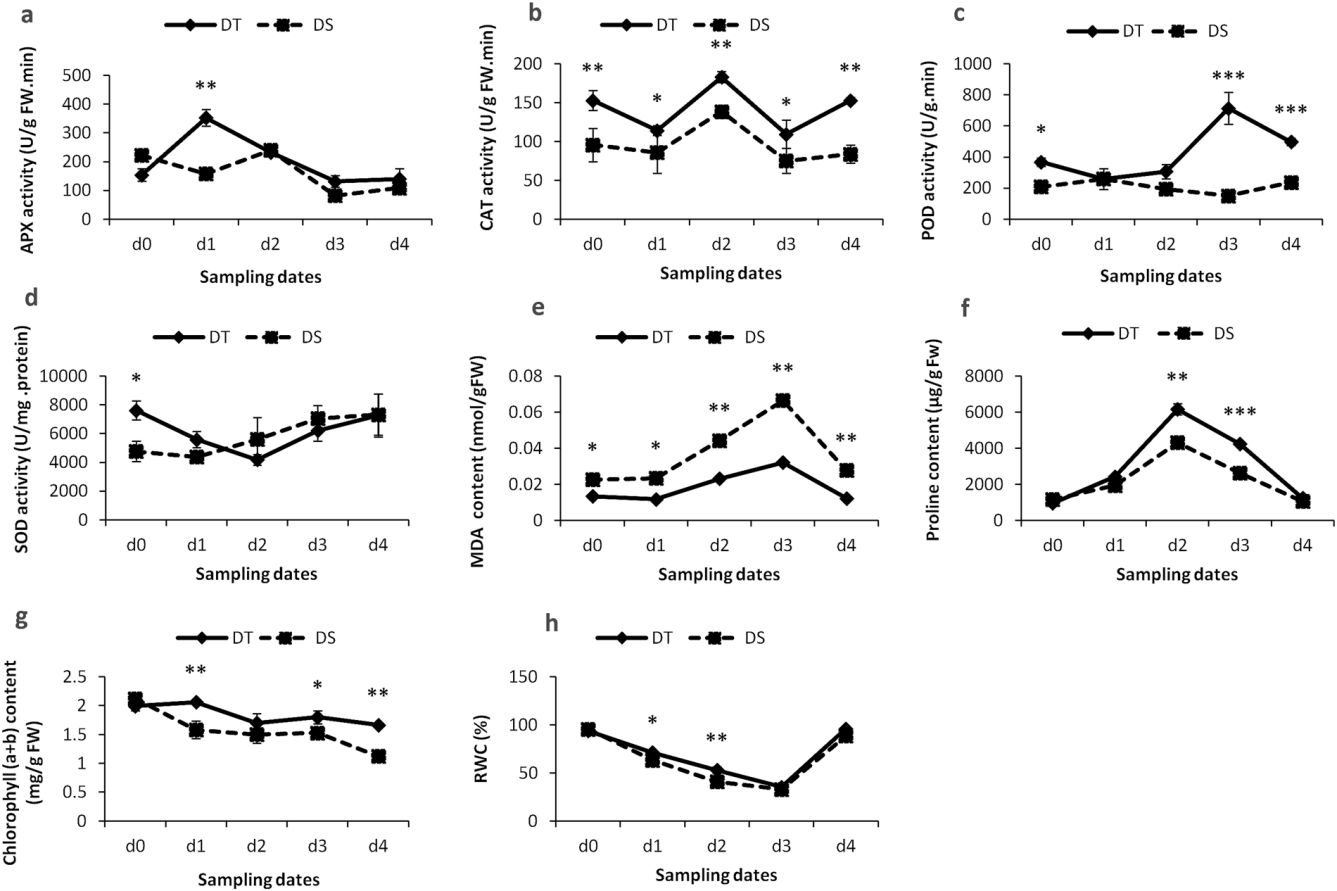
The effect of drought stress on the activity of (**a**) APX, (**b**) CAT, (**c**) POD, (**d**) SOD, (**e**) MDA, (**f**) proline, (**g**) total chlorophyll (a + b), (**h**) RWC, in the leaves of tolerant (DT) and sensitive (DS) sesame genotypes. Means and standard errors based on three replications are indicated. d_0_, d_1_, d_2_, d_3_ and d_4_: plants stressed for 0, 3, 7, 11 days and 3 days of recovery after rewatering, respectively. *, **, *** Significant differences based on Tukey’s multiple range test (P < 0.05, <0.01 and <0.001, respectively).

Prior to drought stress application, it was found that the activity levels of most antioxidant enzymes including SOD, POD and CAT were significantly higher in DT as compared to DS (Fig. 3). Even the leaf MDA content of DS was higher than DT suggesting that DT maintained high levels of antioxidant activity to promptly respond once the stress occurs. During drought periods (d_1_-d_3_), antioxidant enzymes including APX, POD, CAT, and the osmoprotectant proline were in general significantly increased in both genotypes. However, it was obvious that the increase of their activity levels were more striking in DT compared to DS. Only SOD displayed a contrasting pattern in the two genotypes with a continual increase of its activity level in DS while it decreased in DT. The leaf MDA content under stress period increased in the two genotypes but was significantly more accumulated in DS than DT especially at d_3_ when the stress was more intense. POD and proline were the most induced components in the protective machinery at d_3_, hence, may be the most important lines of defense in response to severe drought stress in sesame.

Drought stress has more affected the RWC and total chlorophyll in leaves of DS than DT. After rewatering (d_4_), DT seemed to recover from drought damages sharply than DS as the RWC and the chlorophyll content remained high. Also, the MDA content was still higher in DS while most of the antioxidant enzymes were highly active in DT.

### Transcriptome variability in DT and DS and their relationships in response to progressive water stress and after rewatering

**Figure 4:**
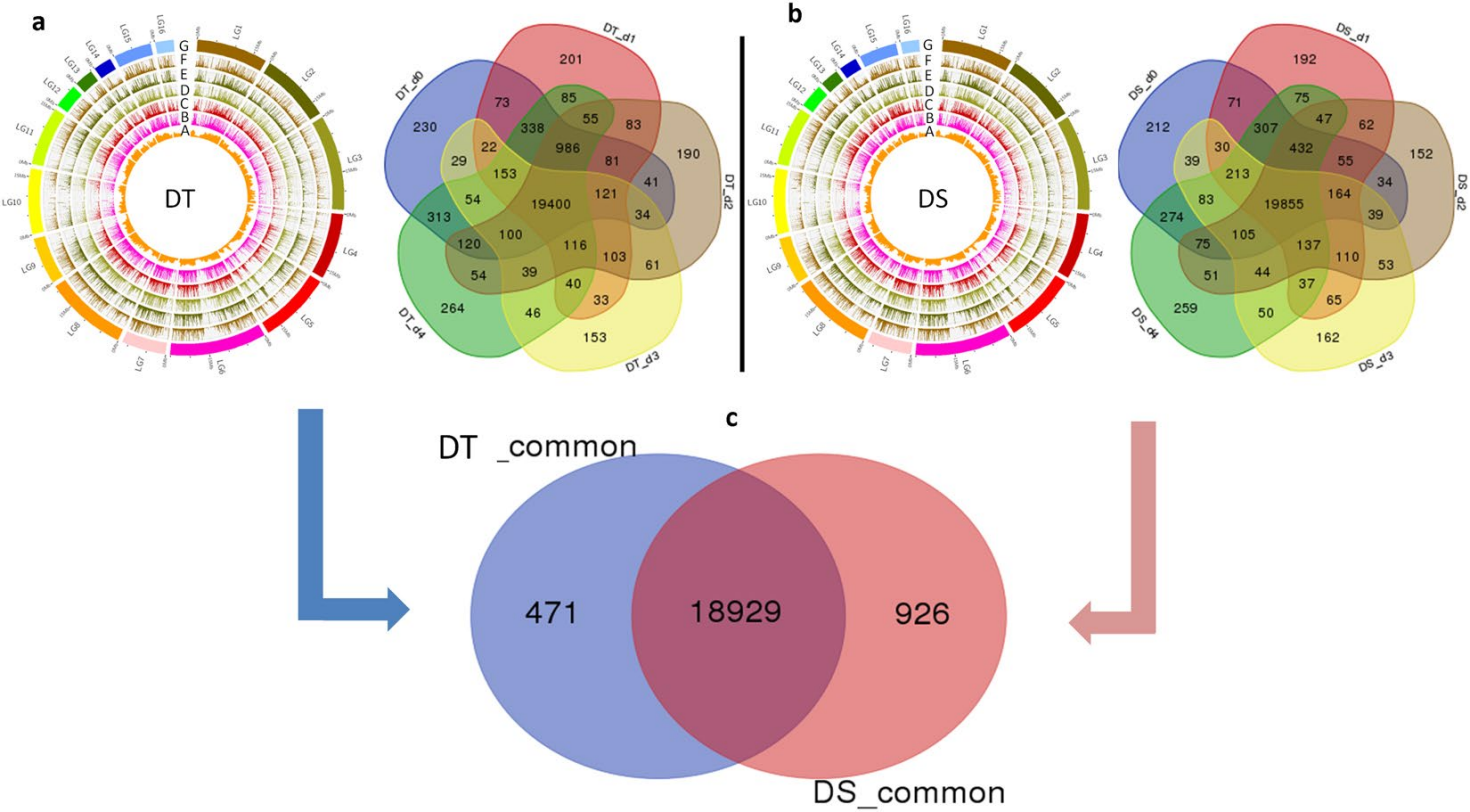
An overview of gene expression (FPKM) and the shared and uniquely expressed gene numbers during a time-point assay of drought stress in (**a**) tolerant (DT) and (**b**) sensitive (DS) sesame genotypes. A, B, C, D and E correspond to the samples collected at d_0_, d_1_, d_2_, d_3_ and d_4_, respectively. F represents the gene density (mRNA, 500-kb window) in the sesame genome and G represents the 16 linkage groups of sesame. (**c**) The commonly shared and uniquely expressed gene numbers during all time-points in DT and DS.

The raw sequences were aligned to the sesame reference genome v.1.0 resulting in approximately 78.68% coverage (Supplementary Table S1). A range of 18,640 to 21,223 genes was expressed at each time point and 1,806 novel genes have been uncovered. Overall, similar numbers of gene were expressed in DS (23,484) and DT (23,618), resulting in a total of 24,113 genes expressed with fragments per kilobase of transcript per million fragments mapped (FPKM) values ranging between 0.01–39,972 (34.8 in average) and 0.01–76,572 (34.7 in average) for DS and DT, respectively (Fig. 4a,b). A total of 19,855 and 19,400 genes were constitutively expressed at all time points in DS and DT, respectively. Of these genes, 18,929 genes were shared by the two genotypes representing 69.7% of the whole sesame genes (Fig. 4c).

**Figure 5:**
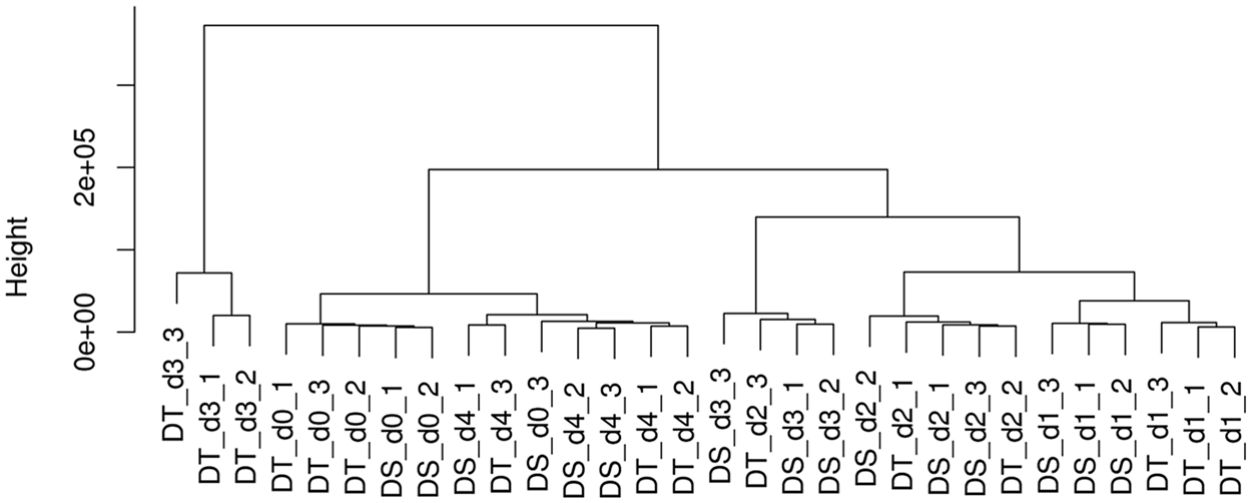
Dendrogram showing correlation among the different samples based on global expression profiles in the drought tolerant (DT) and sensitive (DS) genotypes. d_0_, d_1_, d_2_, d_3_ and d_4_: plants stressed for 0, 3, 7, 11 days and 3 days of recovery after rewatering, respectively.

By comparing the dynamic change of the expressed genes during the different time points, the results showed that the transcriptional responses in sesame root tissue were gradually suppressed by drought with the rate of expressed genes decreasing by 0.9%, 2.3% and 7.2% in DT; and 0.6%, 2.6% and 3.6% in DS at d_1_, d_2_ and d_3_, respectively (Supplementary Figure S1). To investigate the relationship among the transcriptome of different tissue samples (genotype/water availability condition), a correlation analysis was performed on the expression values from all the samples and a dendrogram was generated (Fig. 5). This analysis revealed that sesame transcriptome varied considerably according to the water availability in the soil. Transcriptomes from control plants (d_0_) of DT and DS clustered together with those from plants at d_4_. Likewise, under moderate stress (d_1_ and d_2_) the two genotypes showed closer correlation. However, when the stress becomes more severe (d_3_), DT exhibited divergent gene expression programs compared to DS, which might probably underlie its tolerance to drought stress.

### Dynamic and gene enrichment for the DEGs between sampling dates

**Figure 6:**
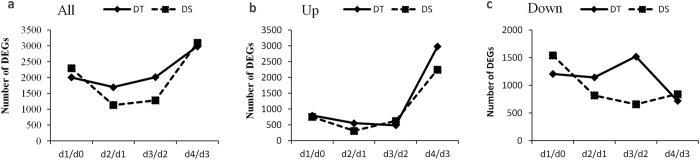
Dynamics of DEGs during time-course of drought stress in DT and DS. (**a**) Variation of all combined DEGs in DT and DS using the previous sampling date as control, (**b**) variation of up-regulated DEGs in DT and DS using the previous sampling date as control and (**c**) variation of down-regulated genes in DT and DS using the previous sampling date as control.

Out of the 24,113 genes expressed in sesame under progressive drought and during recovery, 6,016 genes (24.9%) were significantly and differentially expressed in both genotypes. DS exhibited slightly higher number of DEG (4,970) than DT (4,782) (Supplementary Table S2 and Supplementary Fig. S2). The dynamic of DEGs during the time courses of drought stress and after rewatering was examined by comparing two consecutive time points. The results revealed that the number of DEGs was reduced under stress in both genotypes, but DT was less affected than DS probably owing to its tolerance (Fig. 6).

Gene enrichment analysis of the DEGs demonstrated that early drought stages (d_1_ and d_2_) induced predominantly genes belonging to the category “Metabolism”, further classified into “global and overview maps”, “carbohydrate metabolism”, “amino acid metabolism” and “biosynthesis of other secondary metabolites” based on KEGG pathway annotation database. These genes were mainly involved in “metabolic pathways” and “biosynthesis of secondary metabolites” in both genotypes (Supplementary Fig. S3). Nonetheless, different types of genes were expressed in response to drought stress by the two genotypes and more importantly, DT showed higher DEGs in each pathway. When the stress intensity increases (d_3_), DT and DS displayed different types of DEGs. DT presented DEGs enriched in “biosynthesis of secondary metabolites” and “ribosome” while DS was enriched in “metabolic pathways” and “biosynthesis of secondary metabolites”. However after rewatering, both genotypes displayed same types of DEGs enriched in “metabolic pathways” and “biosynthesis of secondary metabolites”.

GO assignments (biological process, cellular component and molecular function) were used to classify the predicted functions of sesame genes at the different sampling times in both genotypes. Although different sets and numbers of genes were expressed by the two genotypes, similar GO assignments were uncovered: under biological process, the cellular process and metabolic process were prominently represented indicating that some important cellular processes and metabolic activities occurred in sesame roots under stress and during recovery. In the category of cellular component, cell and cell part were the most represented. Catalytic activity and binding were the most abundant among molecular function. However, it is worth to mention that for all GO terms, DT exhibited higher DEGs than DS (Supplementary Fig. S4).

### Core gene set constitutively active in sesame responses to drought stress

A total of 999 and 1,437 DEGs were constitutively expressed from d_1_ to d_3_ in DT and DS, respectively. Of these genes, 722 DEGs (594 down- and 128 up-regulated) were shared by the two genotypes and represent the core gene set involved in sesame response to drought response despite tolerance levels (Supplementary Fig. S5 and Supplementary Table S3). Functional annotation of this core gene set revealed that most of the genes were enriched in molecular functions related to oxido-reductase activity (GO:0016491), antioxidant activity (GO:0016209), catalytic activity (GO:0003824).

### Specific DEGs to DT during drought stress period

The DEGs which were exclusively found in DT were closely analyzed to unravel their functional properties (Supplementary Table S4). At the onset of drought stress, 682 DEGs were present only in DT and these genes were enriched in functions related to interleukin receptor-associated kinase 4/1 (K04733/K04730), cytochrome P450 (K00512), EREBP-like factor (K09286), HSP20 family protein (K13993), solute carrier family 36/15 (K14209/ K14638), glutathione S-transferase (K00799) and superoxide dismutase (K04565). After 7 days of stress (d_2_), we found 411 DEGs solely present in DT. These genes enhancing tolerance to moderate stress were enriched in functions related to HSP20/70 (K13993/ K03283), plant G-box-binding factor (K09060), Myb protein (K09422), 2-hydroxyisoflavanone dehydratase (K13258), solute carrier family 36/15 (K14209/ K14638) and interleukin receptor-associated kinase 4/1 (K04733/K04730). Similarly, when the stress became more intense, 1042 specific DEGs were displayed by DT. This highest number of specific DEGs identified at d_3_ in DT confirmed that a special transcriptional response is crucial for endurance under severe drought. These genes could be classified into 5 majors functional groups including interleukin receptor-associated kinase 4/1 (K04733/K04730), EREBP-like factor (K09286), peroxidases (K00430), mitogen-activated protein kinase 6 (K14512) and pectinesterase (K01051).

### Differential gene expression between DT and DS

**Figure 7:**
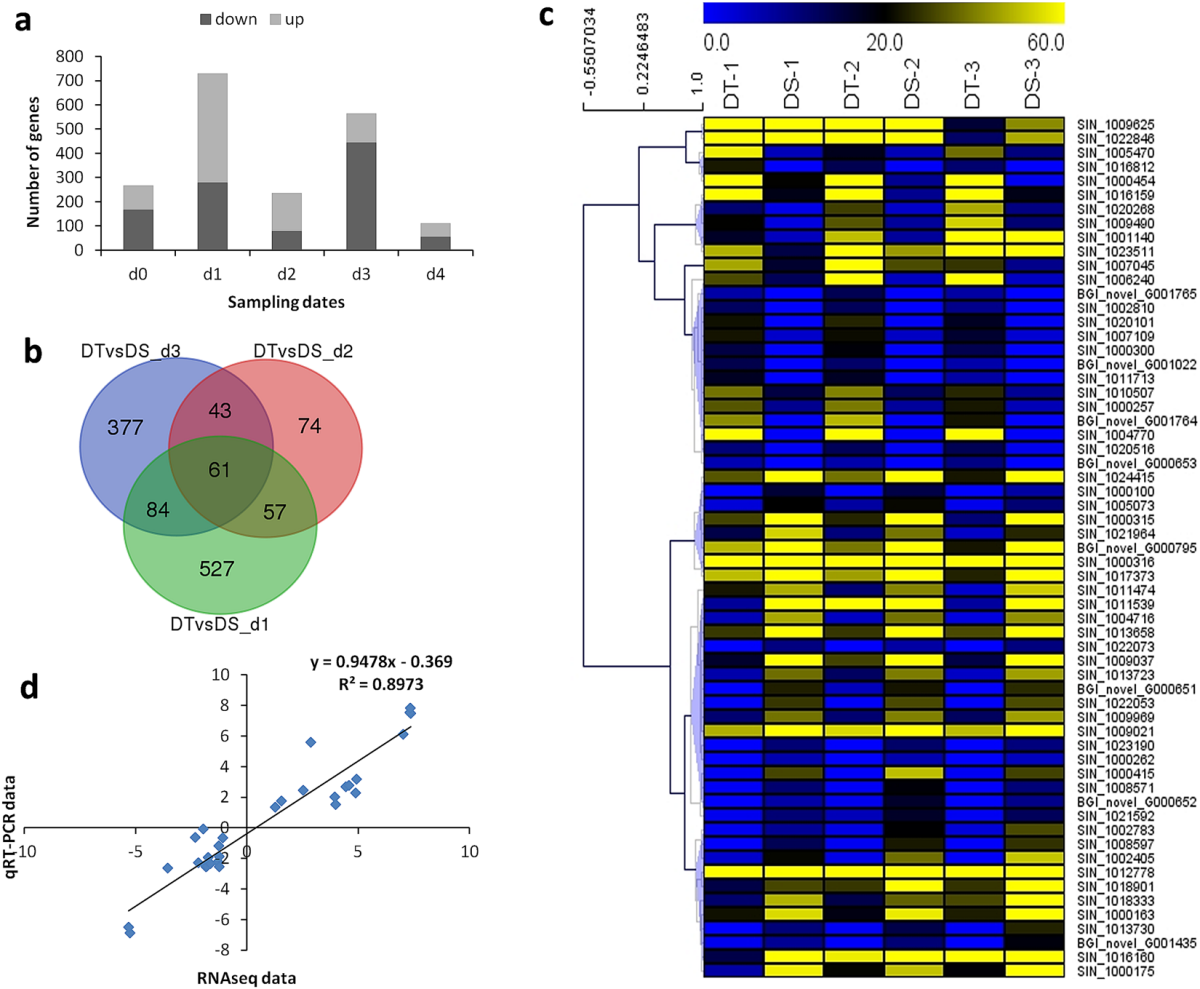
Comparison between DT and DS during drought stress and after rewatering. (**a**) DEGs in DT versus DS at various drought stress time points. (**b**) A venn diagram depicting the common and unique DEGs between d_1_ and d_3_ in DT and DS. (**c**) Expression patterns of the 61 common DEGs between DT and DS under drought conditions. (**d**) Correlation between transcriptome data and qRT-PCR results based on log_2_fold change of 10 selected genes.

The genes imparting higher drought tolerance in sesame were examined by comparing DEGs between DT and DS during drought period. The analysis demonstrated that under stress, many genes (3058) were significantly and differentially expressed between DT and DS (Fig. 7a). During all time points under stress, 61 genes were constitutively, significantly and differentially expressed in DT compared to DS (Fig. 7b and Supplementary Table S5). These genes were highly expressed (FPKM >10 during at least one time point) and may confer higher drought tolerance to DT. They were further classified into 7 groups displaying diverse functions based on hierarchical clustering analysis (Fig. 7c). Most of these genes were NADH dehydrogenase, indole-3-acetate O-methyltransferase, HSP20 family protein, interleukin receptor-associated kinase 4, alpha-L-fucosidase, Cytochrome P450 etc. We selected 10 genes including 5 up-regulated genes and 5 down-regulated genes between DT and DS from the drought tolerance candidate genes and their expression levels were confirmed through qRT-PCR analysis (Supplementary Fig. S6). The qRT-PCR results were strongly correlated with the RNA-seq data (0.90%, Fig. 7d). These results confirmed the high reliability of the RNA-seq data obtained in the present study.

### Gene involved in sesame recovery from drought damage

During recovery from drought stress, DS employed 1,180 DEGs while DT displayed only 442 DEGs. A total of 299 DEGs were shared by the two genotypes suggesting that these genes may be involved in sesame recovery from drought damage (Supplementary Table S6). Go enrichment analysis demonstrated that these genes significantly enhanced biological processes related to catalytic activity (GO:0003824), antioxidant activity (GO:0004871), signal transducer activity (GO:0016209) and metal ion binding (GO:0046872). Based on KEGG database, it was uncovered that the majority of genes involved in sesame recovery from drought were peroxidases (K00430), glutathione S-transferase (K00799), large subunit ribosomal protein L18Ae (K02882) etc. In addition, we identified 143 DEGs which were specific to DT and may help in sesame prompt recovery. These genes fell into 4 majors functional groups viz. protein DEK (K17046), beta-glucosidase (K01188), plasminogen activator inhibitor 1 RNA-binding protein (K13199) and peroxidases (K00430) (Supplementary Table S7).

### Major transcription factors involved in sesame response to drought stress

**Figure 8:**
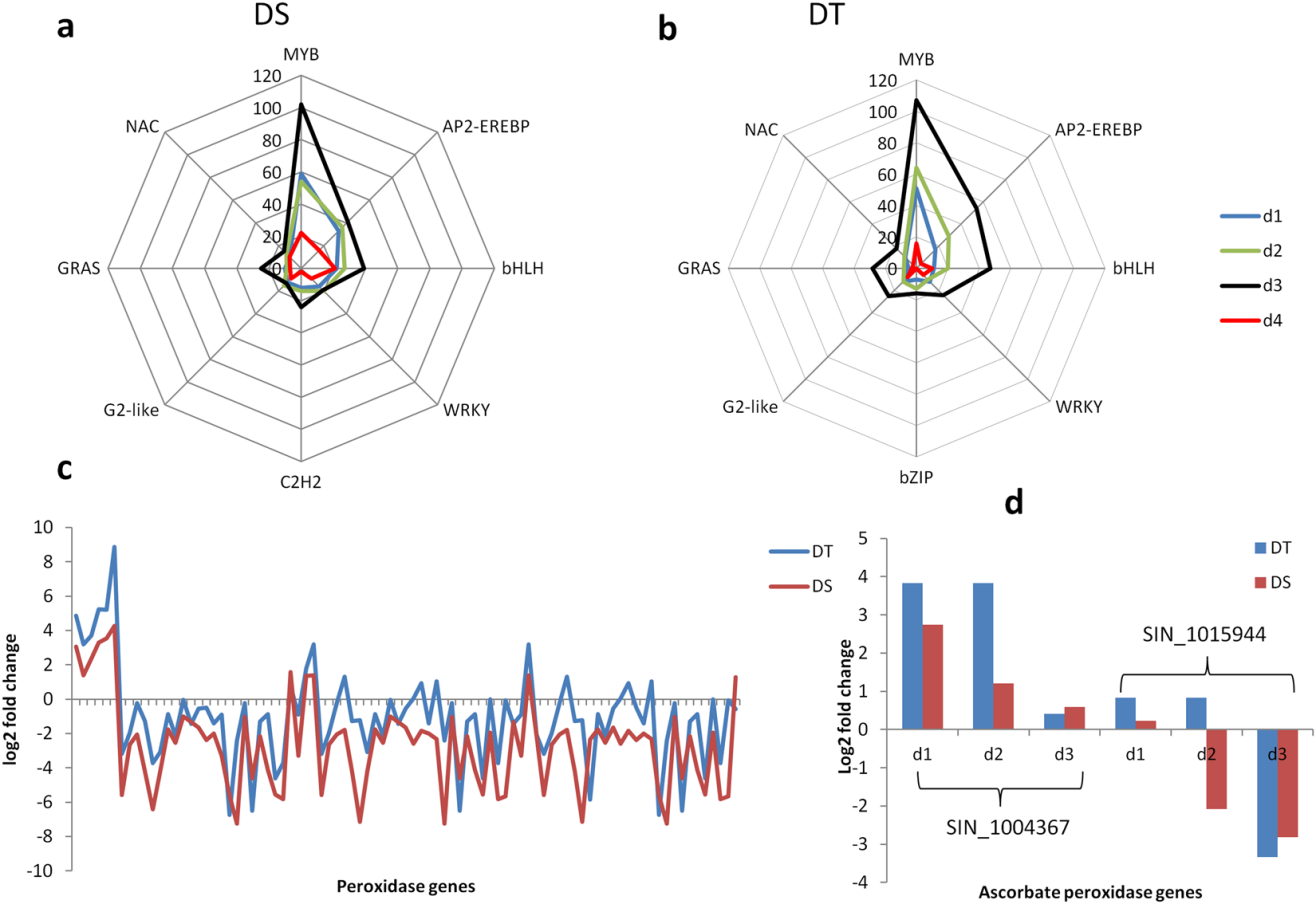
Major transcription factors and pathways involved in sesame drought response. Major transcription factors from DEGs in (**a**) sensitive (DS) and (**b**) tolerant (DT) sesame genotypes, (**c**) log_2_fold change in peroxidase gene expression in DT and DS under drought stress, (**d**) log_2_fold change in ascorbate gene expression in DT and DS under drought. d_0_, d_1_, d_2_, d_3_ and d_4_: data from plants stressed for 0, 3, 7, 11 days and 3 days of recovery after rewatering, respectively.

The dynamic expression of genes involved in transcriptional regulation was surveyed to investigate their role in sesame responses to drought. A total of 2,164 TFs grouped into 46 families were identified among the DEGs. DS displayed a range of 182–432 TFs while DT exhibited a range of 58–496 TFs during all time points. In addition, DS showed higher activity of TFs compared to DT (1,191 vs 973). In general, the number of TFs increased according to the severity of the stress. The members of MYB were the most abundant and active TFs under drought stress in sesame. They were followed by members of AP2-EREBP, bHLH, WRKY, C2H2, bZIP, G2-like, GRAS and NAC types (Fig. 8a,b). These TFs showed diverse expression patterns, indicating that they modulate differently sesame responses to drought stress.

### Role of important phytohormones in sesame response to drought

Three main hormonal signaling pathways, including ABA, Ethylene and Jasmonic acid (JA) were identified as the important hormonal pathways involved in drought stress responses in sesame. DEGs belonging to the Ethylene and ABA signaling pathways were the most abundant and highly expressed ones. In the Ethylene pathway, several components of the biosynthesis and signaling pathway have been identified, such as S-adenosylmethionine synthetase (K00789), aminocyclopropanecarboxylate oxidase (K05933), Ethylene-insensitive protein 2, 3 (EIN2, EIN3), Ethylene receptors and Ethylene response factor (ERF) in both genotypes. ERF were the fourth and fifth most abundant genes in DT and DS, respectively. Moreover, we found that majority of these genes were higher expressed or repressed in DT than DS. Concerning the ABA biosynthesis and signaling pathway, 96 DEGs were identified in DT while only 75 genes were significantly expressed under stress in DS. The most abundant components were the abscisic acid receptor PYR/PYL family (K14496), protein phosphatase 2 C (PP2C) (K14497) and ABA responsive element binding factor (ABF) (K14432). More importantly, all of these genes were up-regulated showing a positive response to drought stress in sesame. Similarly to Ethylene related genes, most of ABA related genes were strongly induced in the tolerant genotype. For example the gene *SIN_1023281* (PP2C) increased by 565 fold its initial activity level during each time of drought stress in the DT but was only increased by 96 fold in DS. Finally, the Jasmonic acid pathway also involved more DEGs in DT (76) than in DS (50). Interestingly, all these genes were down-regulated under stress showing that Jasmonic acid is a negative regulator of drought response in sesame.

### NF-kappa B signaling pathway is implicated in sesame drought response

The most abundant DEGs found during all time points under drought stress in DT and DS were related to interleukin receptor-associated kinase 4/1 (IRAK4/1). These genes belong to the NF-kappa B signaling pathway which control transcription of DNA, cytokine production and cell survival under stress. Most of IRAK1/4 genes were commonly significantly repressed in both genotypes but 46 IRAK1/4 genes were particularly down-regulated exclusively in DT. We infer these genes might play crucial role in the cell maintenance in the root cortex of DT.

### Antioxidant enzymes related genes operating in sesame drought tolerance

Peroxidases (POD) were the most represented antioxidant enzyme in response to drought stress in the two genotypes. In total, DT expressed more DEGs (142) encoding POD enzyme compared to DS (84). Moreover, the induction rate of POD related genes was quite higher in DT which may be a positive feature for its tolerance (Fig. 8c). This result confirmed well the strong activity level of POD enzyme in DT detected based on the biochemical method. Another important antioxidant enzyme involved in drought stress response in plants is superoxide dismutase (SOD). We found 2 types of SOD including Cu-Zn type (K04565) and Fe-Mn type (K04564) in the DEGs. Under stress, SOD related genes were all sharply reduced except for the gene *SIN_1010491* in DT. In contrast, SOD related genes were not significantly affected in DS and *SIN_1010491* were highly induced at d_2_ and d_3_. The expression patterns of SOD related genes in the two genotypes corroborated well the biochemical results. In another realm, three genes *SIN_1015805*, *SIN_1007469*, *SIN_1015806* were found to encode catalase within DEGs of DT and DS. The expression of the gene *SIN_1007469* was strongly affected under stress but the remaining two genes exhibited a positive response to drought with induction rate ranging from 2.3- to 2.7 fold and 1.4- to 1.6-fold their initial expression level in DT and DS, respectively.

Finally, we searched for the genes encoding ascorbate peroxidase enzyme (APX) in the DEGs of the two genotypes and discovered 2 main genes: *SIN_1004367* and *SIN_1015944*. These genes showed similar patterns of expression in DS and DT but the expression levels were more striking in DT, exactly as found based on biochemical dosage of APX (Fig. 8d).

### Osmoprotective related genes functioning in drought responses

A rapid and reversible increase in the intracellular concentration of free proline to very high values is essential for plant endurance under stress. Here, we found within DEGs of both genotypes, only one gene *SIN_1023484* (pyrroline-5-carboxylate reductase (P5CR)) which catalyses the final step of proline synthesis. *SIN_1023484* was continuously up-regulated in DT by 1.5-, 2.5-, and 2.8- fold its initial expression levels at d_1_, d_2_ and d_3_, respectively. In the sensitive genotype, this gene was first down-regulated at d_1_ by 0.6-fold before significantly induced at d_2_ and d_3_ by 1.5- and 2-fold, respectively. These results demonstrate that DT accumulated higher proline related mRNA and this may be the basis of its higher concentration of proline under stress. Trehalose is another important osmolyte which protects cell membrane from decay under abiotic stresses. A total of 8 DEGs were commonly and significantly involved in the occurrence of trehalose and trehalose biosynthesis pathway in DT and DS. In DS, three genes encoded for trehalose 6-phosphate synthase (TPS) and only one gene (*SIN_1023896*) has increased its expression level (by 3- fold the initial level). We uncovered 4 genes playing the same function in DT including *SIN_1023896* up-regulated by 9-fold and a unique DEG to DT (*SIN_1011804*), which increased its expression by 8 -fold the initial level. In the other hand, genes functioning as trehalose 6-phosphate phosphatase (TPP) and glucose-1-phosphate adenylyltransferase did not display conspicuous difference in gene expression patterns between the two genotypes.

Glycine betaine is a quaternary ammonium compound well known as compatible solute which occurs abundantly in response to dehydration stress in plants. By inspecting DEGs related to the glycine betaine biosynthesis pathway, we detected 2 main components including choline dehydrogenase (K00108) and betaine-aldehyde dehydrogenase (K00130) in the two genotypes. Under short term drought period, the genes *SIN_1016595* and *SIN_1013006* (choline dehydrogenase) were induced in both genotypes but more strongly in DS. When the stress became severe, all the genes related to this pathway were down-regulated in DT and DS. In contrast to the different pathways analysed so far, genes involved in the glycine betaine biosynthesis pathway seem to be less affected in the sensitive genotype than in the tolerant and this may suggest that the glycine betaine biosynthesis pathway is not cardinal for drought survival in sesame.

## Discussion

Root represents the first plant organ that detects a limitation of the water supply in the soil and a defective root system due to damages from drought stress impairs plant survival^27^. Cortical parenchyma serves as a storage area of nutrients and water^28^. Air spaces in cortical tissue due to collapsed cells could interrupt radial water movement in roots and reduce root/soil contact because of root shrinkage away from the soil thus limiting water uptake in drying soils^29^. In contrast to DS, in our study we found that DT relied on a well-functioning root under stress facilitating water availability for the maintenance of its aboveground tissues. Hence, RWC was higher in DT which has been reported to be associated with higher level of photosynthetic pigments, membrane stability index, osmolytes and antioxidant activities^30^. We found that the antioxidative machinery was more effective in DT than DS during and after stress period especially POD which was more active under severe stress. The higher content of malondialdehyde in DS further confirms its weak ability for ROS detoxification that lead to cortical cell decay and heavy plant wilting. We deduced that DS displays a less efficient “first line” of defense against ROS in condition of drought stress and therefore is exposed to a more severe oxidative stress. Fazeli *et al*.^22^, Kadkhodaie *et al*.^31^ also concluded that the greater increase of antioxidant enzymes in leaves were related to drought tolerance of sesame genotypes. Beyond the enzymatic system, plants also accumulate some organic osmolytes mainly glycine betaine and proline in response to environmental stresses such as drought, salinity, extreme temperatures^2^, etc. Proline plays a pivotal role in plant cytoplasmic osmotic adjustment under drought, stabilizing cell membranes thereby preventing electrolyte leakage and regulating concentrations of ROS within a normal range^32^. In this study, proline accumulation was significantly higher in DT compared to DS especially when drought stress became intense. It is inferred that a high accumulation of proline improves drought tolerance in sesame as described in other plant species^32, 33^.

To grasp the genomic background underlying sesame responses to drought stress, temporal transcriptome data have been generated and scrutinized in DT and DS. The expressed genes in DT and DS are quite higher than previous transcriptome reports in sesame^34, 35^. Globally, drought stress gradually alters gene expression in sesame^36^. Gene activities were at the lowest level after 11 days of water deprivation in the two genotypes and this may imply that a longer drought period would elicit a stronger alteration of gene expression^37^. In the other hand, it appears that a severe water deficit triggers adapted transcriptional responses in DT contrary to DS. For instance, at d_3_, DT showed DEGs enriched in ribosome pathway which was absent in DS according to KEGG pathway analysis. Ribosomes are organelles that synthesize proteins (translation) and plants can respond to environmental changes with various mechanisms occurring at transcriptional and translational levels. By comparing ribosome profiling and RNA-seq data of maize seedlings under drought conditions, Lei *et al*.^38^ demonstrated that fold changes of gene expression at transcriptional level moderately correlate with that of translational level, meaning that drought stress can induce transcriptional and translational responses independently. The high activity of genes related to ribosome pathway in DT at d_3_ suggests that the tolerant genotype responds to severe drought stress with highly translational mechanisms acting synergistically with that of transcription.

Some DEGs were continuously expressed under drought stress in the two genotypes. These genes represent the core gene set in sesame that functions in response to drought stress, despite tolerance levels. It was found that many of these genes are peroxidases encoding genes. Peroxidases are involved in the defense against abiotic stresses by means of their role in the detoxification of H_2_O_2_^39^. Roldán *et al*.^40^ revealed that, total peroxidase activities were crucial for drought performance of *Juniperus oxycedrus* seedlings. In addition, Cytochrome P450 encoding genes were also predominant in this core gene set. Cytochrome P450s are among the largest protein coding gene families in plant genomes. In rice, the mutation of *CYP96B4/SD37*, a gene coding for a cytochrome P450 enhanced drought tolerance^41^. Also, Nam *et al*.^42^ discovered that transgenic rice which overexpresses *AtCYP78A7* exhibited changes in amounts of metabolites which may assist in improving drought tolerance by playing crucial roles in stress-responsive pathways including GABA biosynthesis, sucrose metabolism and antioxidant defenses. Another major gene family uncovered in the core gene set was the aquaporin. Aquaporins are thought to be involved in plant adaptation to drought stress^43, 44^. They facilitate the diffusion of water molecules across membranes according to a water potential gradient. Beyond this core gene set, we also discovered that DT displayed unique DEGs highly expressed during different time points under stress. Several important drought-related functional genes including peroxidases, pectinesterase, solute carrier family 15, interleukin receptor-associated kinase, HSP20/70 and EREBP were detected suggesting that these genes probably constitute the basis of drought tolerance features in DT^45, 46^. MYB and AP2-EREBP genes were the major transcription factors implicated in response to drought and were found in more abundantly in DT. Previously, Dossa *et al*.^20^ also reported that the DREB subfamily genes related to the AP2-EREBP were highly active in short term drought response in sesame. More importantly, MYB and DREB genes have been demonstrated to be major ingredients of the ABA pathway^47, 48^. The involvement of ABA in mediating drought stress has been extensively studied and it is well established that ABA plays crucial role in regulating water status through guard cells and growth as well as by induction of genes that encode enzymes and other proteins involved in cellular dehydration tolerance^49, 50^. Analysis of phytohormones in sesame denoted that ABA seconded by Ethylene, were the most important hormonal pathways positively involved in drought responses of sesame. Likewise, by inspecting DEGs related to antioxidant enzymes and osmoprotectants, our transcriptome results confirmed that DT not only strongly induced these key genes but also invoked more DEGs which impart drought tolerance. The transcriptome results support well the striking antioxidant enzyme activities and the strong accumulation of proline detected in DT through biochemical dosage. Comparison of gene expression between DT and DS under stress period unveiled 61 genes highly, differentially and continuously expressed, indicating that they may impart higher drought tolerance to DT. Therefore, further studies may focus on these genes to characterize them and find out the favorable alleles conferring high drought tolerance in sesame.

After rewatering, gene expression was resumed in both sesame genotypes as mentioned in other crops^36, 51^. Transcripts coding for peroxidases were also found in abundance in DEGs involved in recovery from drought. Glutathione *S*-transferase (GSTs, EC 2.5.1.18) is another gene family playing similar function like peroxidases. In both animals and plants, GSTs are induced by diverse environmental stimuli, with increased levels used to maintain cell redox homeostasis and protect organisms against oxidative stress^52^. All GST genes were found up-regulated after rewatering indicating that they act positively to alleviate drought damage in sesame as demonstrated in Arabidopsis^53^.

In summary, we conclude that under drought stress and during recovery, the tolerant genotype sparks efficient transcriptional responses enriched in signal perception, transduction, antioxidant genes especially peroxidases, osmoprotectants and cell wall integrity maintenance which activities significantly increased in roots according to the severity of the stress. Fundamental genes in these pathways play adaptive roles in mediating osmotic adjustment, ROS scavenging and protecting cellular structures in the stressed plants. As a consequence, ROS detoxification was highly effective in DT thus allowing the plant to keep maximum of its root cortical cells which are a critical reservoir of useful water under stress. Water was then available and mobile to the aboveground tissues thanks to the high activity of aquaporin genes, supporting the photosynthetic activity and improving plant endurance under drought stress. In opposition to DS, DT engaged two key strategies to sustain drought stress: the first is to implicate high number of DEGs related to pivotal pathways responsible of drought stress responses and damage alleviation; secondly, it has higher ability to potently express these key genes which help to efficiently combat the stress. The mechanism by which DT could higher induce genes as compared to DS is still unclear. We speculate that some genetic variants existing in these key genes may lead to their over-expression in DT. Wherefore, future experiments such as genome-wide association studies in a large panel could better deepen our insight into the genetic architecture of drought tolerance in sesame.

## Methods

### Materials and stress treatment

Seeds of two sesame accessions with different tolerance levels to drought [ZZM0635-drought tolerant (DT)] and [ZZM4782-drought sensitive (DS)] were obtained from a large collection of sesame accessions preserved at the China National Genebank, Oil Crops Research Institute, Chinese Academy of Agricultural Sciences^24^. Plants were sown during raining season (June 11^st^ 2016) in pots (25 cm diameter and 30 cm depth) containing 7 Kg of loam soil with known physicochemical properties mixed with 10% of added compound fertilizer. Experiment was conducted in the experimental field of the Oil Crops Research Institute (Wuhan, China). The seedlings were thinned at 2 true leaves stage and 3 plants per pot were kept under natural conditions with mean temperature 31/27 °C day/night during this stage. The experiment was carried out under a completely randomized split-plot with 6 replicates. The two genotypes and the five drought treatments were arranged as main-plot and sub-plot, respectively. With 3 plants per pot, each treatment therefore involved 18 plants. Seedlings were well watered to keep optimal soil moisture conditions [35% volumetric water content (vwc)]. The soil moisture expressed as vwc was measured manually in each pot using a Moisture Meter *Takeme* (China) over the entire experiment. The water stress treatment was imposed at the early anthesis [47 days after sowing (DAS)], and pots were moved to a greenhouse to avoid rainy days interfering [sampling date 1 (d_0_)]. The mean temperature during this stage was 35/30 °C day/night. The moisture gradually decreased by withholding water supply and at 50 DAS the first signs of leaf wilting appeared with soil moisture reaching 15% vwc [sampling date 2 (d_1_)]. When the moisture reached 9% vwc [54 DAS corresponding to the sampling date 3 (d_2_)], 6% vwc [58 DAS corresponding to the sampling date 4 (d_3_)], plants showed mild-wilting to critical wilting levels. At 58 DAS, watering was resumed for 4 days to reach 35% vwc, corresponding to the re-watering phase [sampling date 5 (d_4_)]. A schematic sketch of the experiment is presented in Fig. 1. At each of the above-indicated sampling dates, materials from three independent plants of the same pot (three biological replicates of leaf and root samples) were collected for RNA extraction, biochemical and physio-anatomical analyses.

### RNA extraction and transcriptome sequencing

Total RNA was extracted according to protocol described by Wei *et al*.^54^. After extraction, contaminating genomic DNA was removed by DNase I (Qiagen, Hilden, Germany) treatment and Oligo (dT) was used to isolate mRNA. The mRNA was mixed with the fragmentation buffer. Quantity and quality of mRNA were assessed by ND-1000 Nanodrop spectrometer (Nanodrop Technologies, USA) and on 2% denatured agarose gel. Next, the cDNA was synthesized using the mRNA fragments as templates. Short fragments were purified and resolved with EB buffer for end reparation and single nucleotide A (adenine) addition. After that, the short fragments (200 ± 25 bp) were ligated with adapters and the suitable fragments were selected for the PCR amplification. During the QC steps, Agilent 2100 Bioanaylzer and ABI StepOnePlus Real-Time PCR System were used in quantification and quality check of the sample library. The 10 cDNA libraries generated from RNA samples isolated from the roots of DT and DS at d_0_, d_1_, d_2_, d_3_ and d_4_ were paired-end sequenced using Illumina HiSeq. 4000.

### Sequencing reads filtering and genome mapping

The sequencing reads containing low-quality (containing >10% bases with a Phred quality score <20), more than 5% ambiguous residues (N) and adaptor sequences were removed before downstream analyses using FastQC (http://www.bioinformatics.babraham.ac.uk/projects/fastqc/). After filtering, the remaining reads were called “clean reads” and stored in FASTQ^55^. The clean reads were then mapped to the sesame reference genome using HISAT^56^.

### Novel transcript prediction, gene expression analysis and differentially expressed gene detection

After genome mapping, the program StringTie^57^ was used for transcript assembly. Cuffcompare, a tool of cufflinks, was applied on the genome annotation information to identify novel transcripts in the samples^58^. The software program CPC^59^ was employed to predict coding potential of novel transcripts and we further merged them with the reference transcripts to get the complete reference. Next, the clean reads were mapped to the complete reference using Bowtie2^60^. RSEM package^61^ was used to calculate gene expression level for each sample expressed as fragments per kilobase of transcript per million fragments mapped (FPKM). Circos program^62^ was employed to represent the gene expression levels in DT and DS. The differentially expressed genes (DEG) were detected as described by Tarazona *et al*.^63^ based on the parameters: Fold change > = 2.00 and Probability > = 0.8.

### Functional annotation and enrichment analysis

Blast homology searches and sequence annotation were carried out using Blast2GO tool v.3.1.3 (http: //www.blast2go.org)^64^. With the GO annotation result, the DEGs were classified according to the official classification, and GO functional enrichment was conducted using *phyper*, a function of R (with False Discovery Rates (FDR) <0.001 defined as significant enriched). Similarly, KEGG (Kyoto Encyclopedia of Genes and Genomes) pathways were assigned to the assembled sequences using the online KEGG Automatic Annotation Server (KAAS) (http://www.genome.jp/kegg/kaas) and enrichment analysis was also performed based on the same criteria described above.

### Transcription Factor prediction of DEGs

To identify transcription factors (TF) in the DEGs, the software package getorf^65^ was used to find ORF of each DEG. After, ORFs were aligned to TF domains from PlntfDB^66^ (http://plntfdb.bio.uni-potsdam.de/v3.0/index.php?sp_id=ATH) using hmmsearch^67^.

### Validation of gene expression using qRT-PCR

qRT-PCR analyses of target genes were performed according to Dossa *et al*.^20^ using a Light Cycler 480 II (Roche, Switzerland). The relative expression levels of the selected genes normalized to the expression level of *actin7* were calculated from cycle threshold values using the 2^−∆∆Ct^ method^68^. This experiment was carried out in three independent biological replicates and three technical replicates of each biological replicate. The list of primers is presented in Supplementary Table S8.

### Root anatomy

To investigate root anatomical features, samples were taken from the top 4–6 cm (2 cm length) along the taproot and stored in formalin–alcohol–glacial acetic acid (90:5:5, v/v/v) for at least 24 h. Dehydration, staining and transverse sections were processed following descriptions of Wei *et al*.^39^. Images of the root sections were acquired with a Leica scanner Aperio CS2 (Leica Biosystems, Germany) and analysed with the Aperio Imagescope software (Leica Biosystems, Germany).

### Relative Water Content (RWC)

RWC was calculated based on the formula RWC (%) = (FW − DW)/(TW − DW) × 100, where: FW is the fresh weight of the open second leaf, TW is the turgid weight of the second leaf, which was incubated in distilled water for 6 h in laboratory room temperature, and DW is the dry weight of the leaves after 24 h in an oven at 60 °C. Three plants from the same pot were used for RWC analysis, which resulted in three biological replicates for each genotype and sampling date.

### Measurement of protective enzyme activities in leaf samples

The extract for all antioxidant enzymes was prepared as described by Chakraborty *et al*.^69^. All experiments were performed with three biological and three technical replicates.

Ascorbate peroxidase (APX, EC 1.11.1.11) was assayed by recording the decrease in optical density due to ascorbic acid at 290 nm in a spectrophotometer (Biomate^TM^ 3 S Spectrometer, USA) according to method described by Nakano and Asada^70^. Peroxidase (POD, EC 1.11.1.7) activity was measured as described by Abeles and Biles^71^. The rate of benzidine oxidation was measured at 470 nm. Total superoxide dismutase (SOD, EC 1.15.1.1) activity was estimated by the inhibition of the photochemical reduction of nitroblue tetrazolium (NBT) by the enzyme^72^. The absorbance was recorded at 560 nm and one unit of enzyme activity was defined as the amount of SOD that produced 50% inhibition of NBT reduction % in comparison with tubes lacking enzyme. Catalase (CAT, EC 1.11.1.6) activity was assayed by measuring the decrease in absorbance of H_2_O_2_ at 240 nm as a consequence of H_2_O_2_ consumption^73^.

### Free proline, malondialdehyde and chlorophyll contents in leaf samples

Free leaf proline content was determined according to method of Bates *et al*.^74^ with slight modifications. The concentration of proline was calculated using a standard curve. Lipid peroxidation was determined by measuring the amount of malondialdehyde (MDA) formation using the thiobarbituric acid (TBA) reaction, as described by Heath and Packer^75^. The absorbance of supernatant was measured at 532 nm. The value for non-specific absorption at 600 nm was subtracted. The concentration of MDA was calculated by using an extinction coefficient of 155 mM^−1^cm^−1^. The total chlorophyll (a + b) content was determined following description of Wei *et al*.^39^. The absorption values of the supernatants were measured at 665 nm, 649 nm, and 470 nm, and chlorophyll contents were calculated according to the formula of Lichtenthaler and Wellburn^76^.

### Statistical analysis

Statistical analysis was conducted using Minitab® and significance of the treatment differences was assessed by a one-way analysis of variance (ANOVA) followed by Tukey’s multiple range test (P < 0.05).

### Data Availability

The RNA-seq datasets supporting the results of this article were submitted to GenBank (BioSample IDs: SAMN06130606 and SAMN06130607), accessible at http://www.ncbi.nlm.nih.gov/sra/?term=SRP095661.

## Electronic supplementary material

Supplementary Table S1

Supplementary Table S2

Supplementary Table S3

Supplementary Table S4

Supplementary Table S5

Supplementary Table S6

Supplementary Table S7

Supplementary Table S8

Supplemenatry materials
