# Supplementary material for: Transcriptomic, biochemical and physio-anatomical investigations shed more light on responses to drought stress in two contrasting sesame genotypes
Source: Sci Rep. 2017 Aug 18;7:8755. doi: 10.1038/s41598-017-09397-6 (PMC5562740; doi:10.1038/s41598-017-09397-6)

Transcriptomic, biochemical and physio-anatomical investigations shed more light on responses to drought stress in two contrasting sesame genotypes

Komivi Dossa[1,2,3], Donghua Li[1], Linhai Wang[1], Xiaomin Zheng[4], Aili Liu[1], Jingyin Yu[1], Xin Wei[1], Rong Zhou[1], Daniel Fonceka[2,5], Diaga Diouf[3], Boshou Liao[1], Ndiaga Cissé[2] & Xiurong Zhang[1]

Supplementary Figures

Supplementary Figure S1. Dynamics of expressed genes during time-course of drought stress and after rewatering in DT and DS

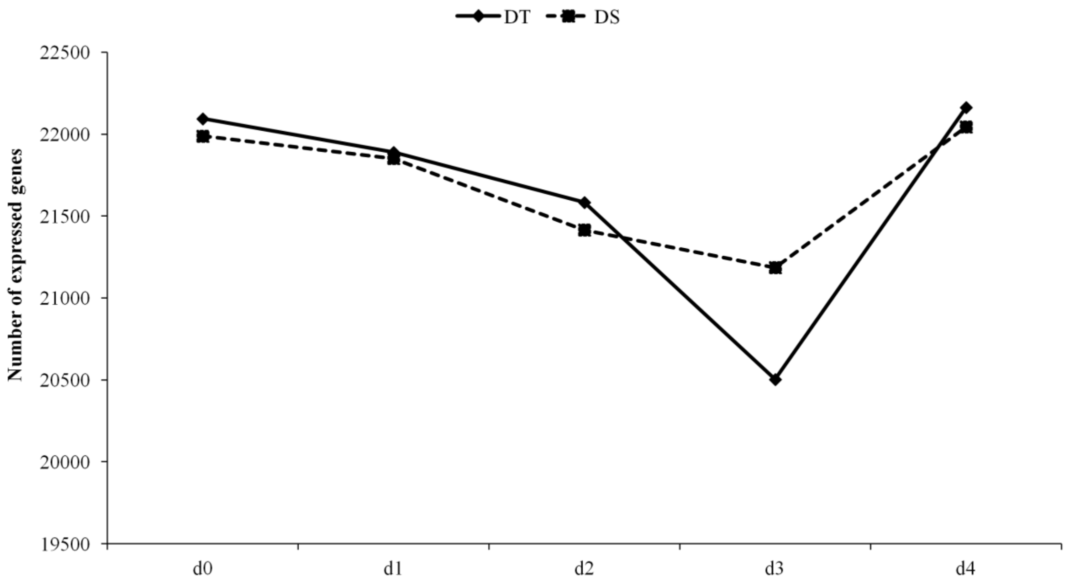

Supplementary Figure S2. Volcano plot of DEGs in DT and DS during time-course of drought stress and after rewatering with d_0 as control

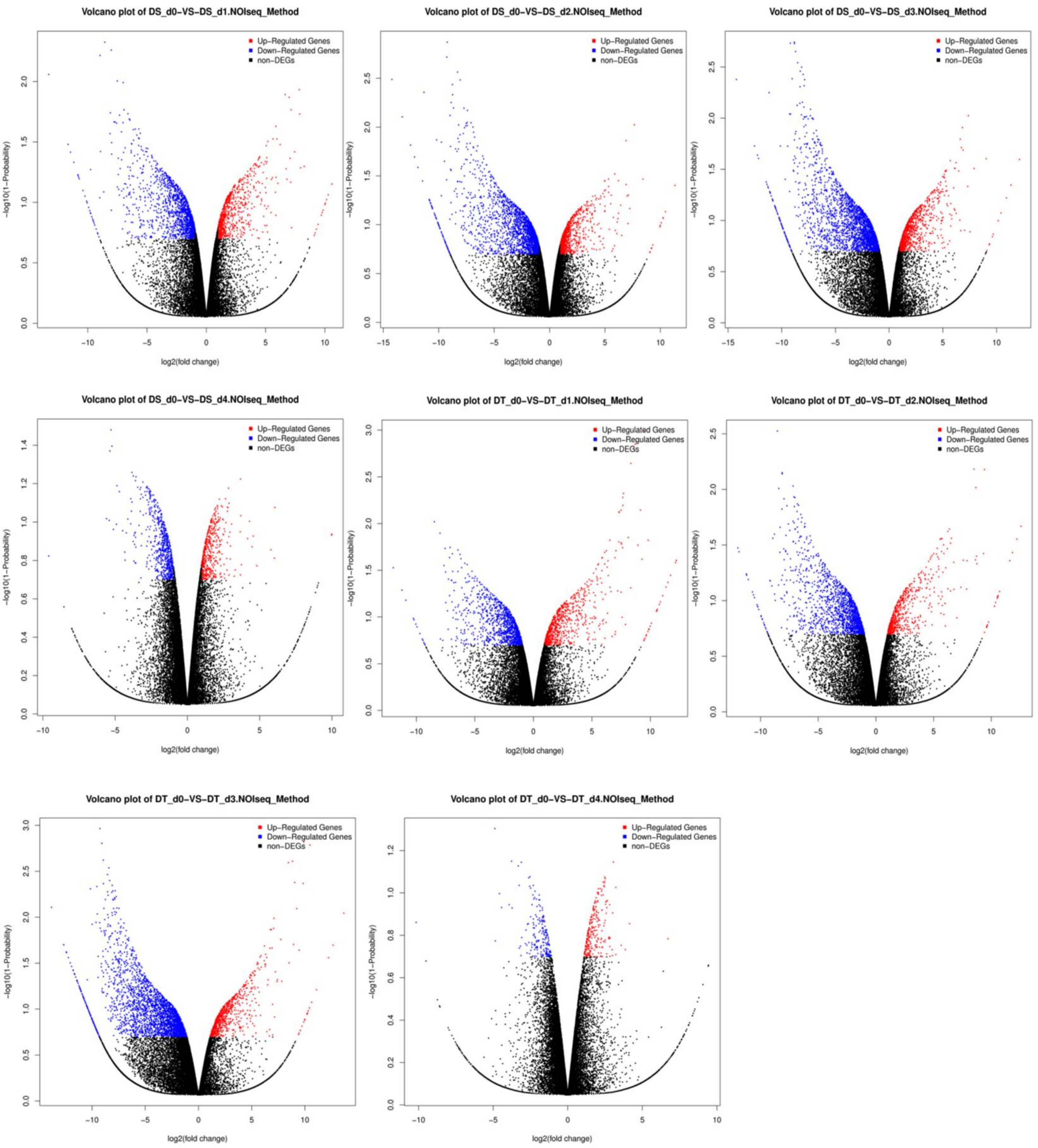

Supplementary Figure S3. KEGG pathway enrichment of DEGs in DT and DS during time-course of drought stress and after rewatering

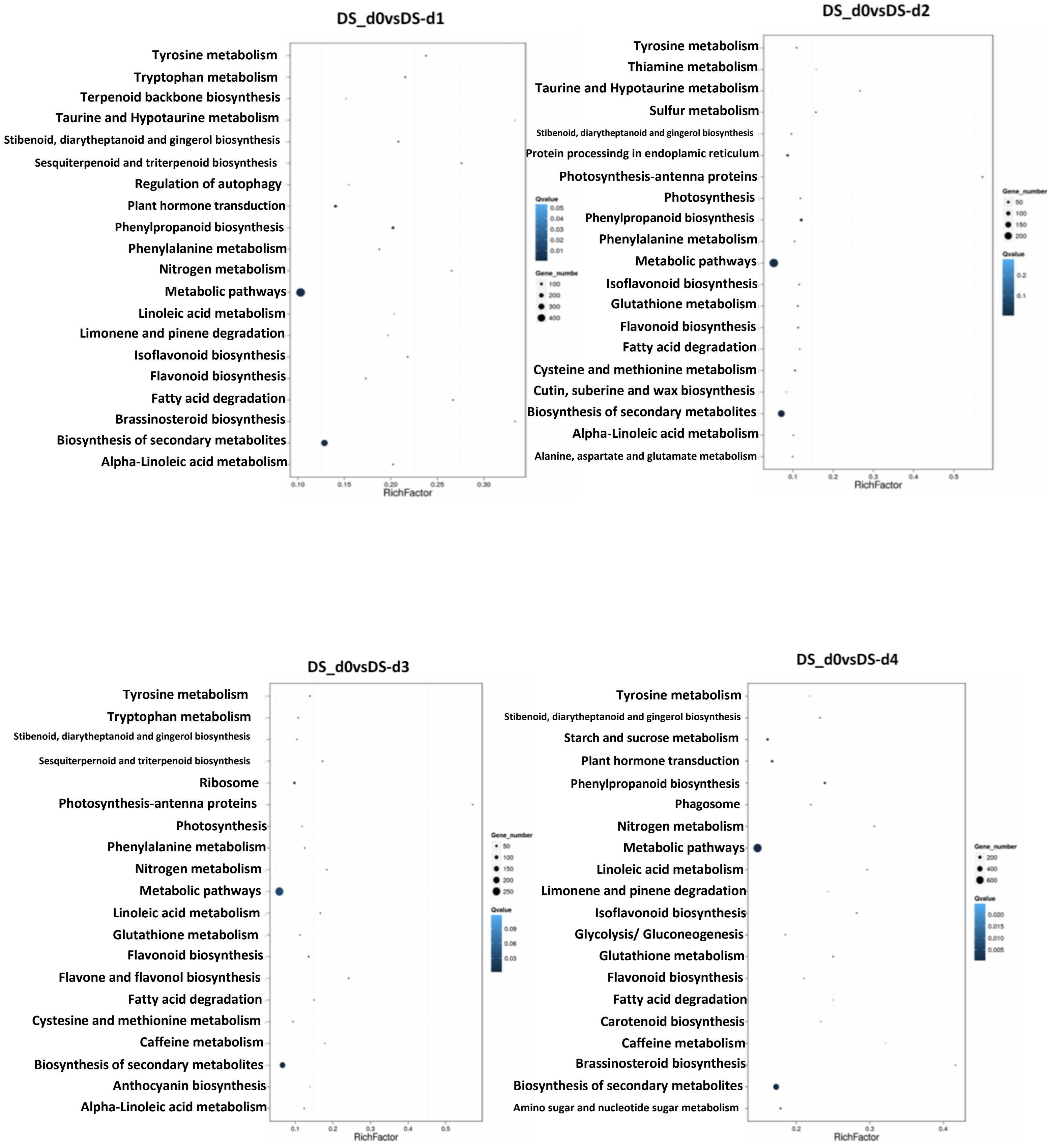

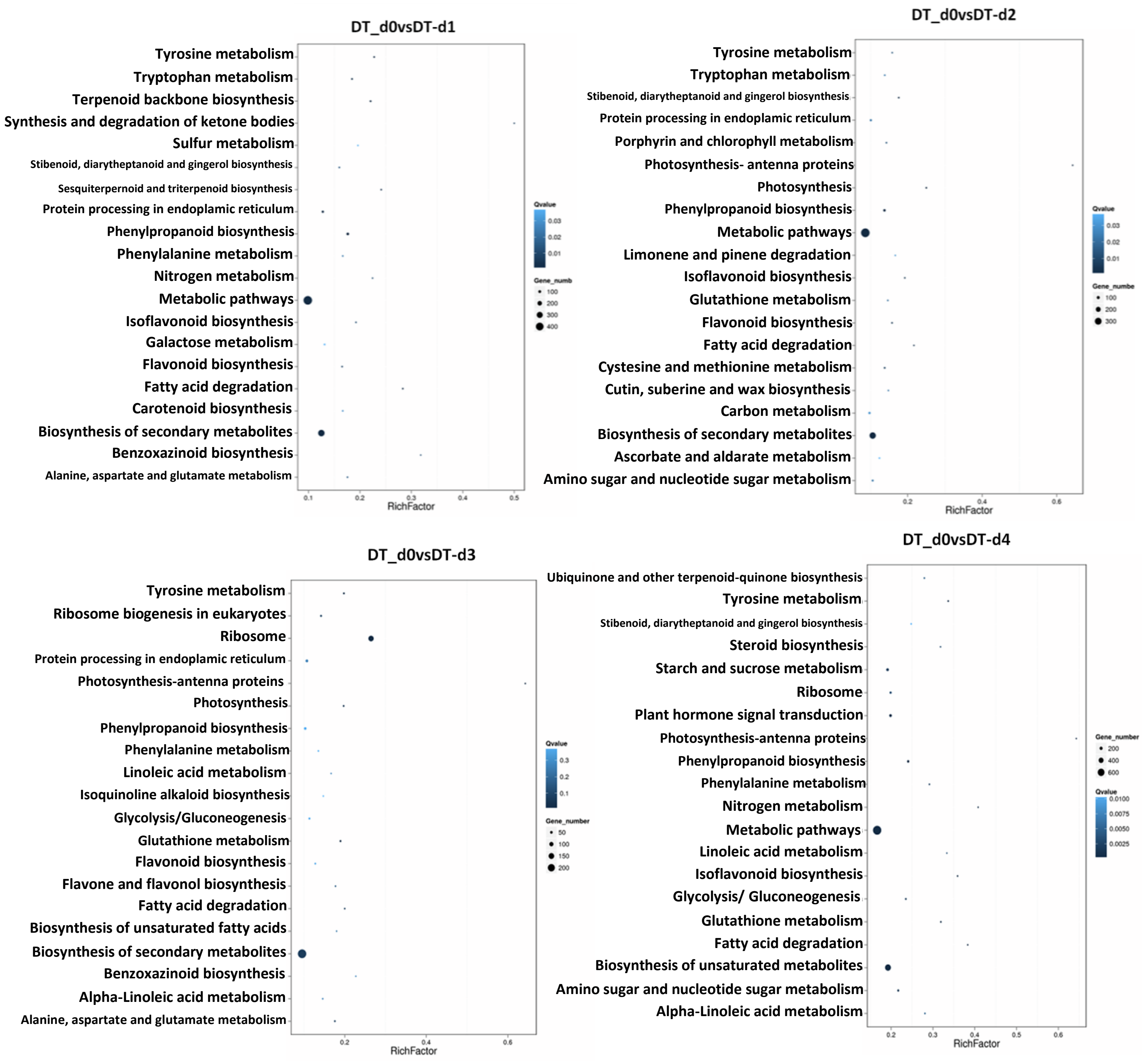
DT_d0vsDT-d1
Tyrosine metabolism
Tryptophan metabolism
Terpenoid backbone biosynthesis
Synthesis and degradation of ketone bodies
Sulfur metabolism
Stibenoid, diarytheptanoid and gingerol biosynthesis
Sesquiterpernoid and triterpenoid biosynthesis
Protein processing in endoplamic reticulum
Phenylpropanoid biosynthesis
Phenylalanine metabolism
Nitrogen metabolism
Metabolic pathways
Isoflavonoid biosynthesis
Galactose metabolism
Flavonoid biosynthesis
Fatty acid degradation
Carotenoid biosynthesis
Biosynthesis of secondary metabolites
Benzoxazinoid biosynthesis
Alanine, aspartate and glutamate metabolism
RichFactor
Qvalue
Gene_numb
DT_d0vsDT-d2
Tyrosine metabolism
Tryptophan metabolism
Stibenoid, diarytheptanoid and gingerol biosynthesis
Protein processing in endoplamic reticulum
Porphyrin and chlorophyll metabolism
Photosynthesis- antenna proteins
Photosynthesis
Phenylpropanoid biosynthesis
Metabolic pathways
Limonene and pinene degradation
Isoflavonoid biosynthesis
Glutathione metabolism
Flavonoid biosynthesis
Fatty acid degradation
Cysteine and methionine metabolism
Cutin, suberine and wax biosynthesis
Carbon metabolism
Biosynthesis of secondary metabolites
Ascorbate and aldarate metabolism
Amino sugar and nucleotide sugar metabolism
RichFactor
Qvalue
Gene_numbe
DT_d0vsDT-d3
Tyrosine metabolism
Ribosome biogenesis in eukaryotes
Ribosome
Protein processing in endoplamic reticulum
Photosynthesis-antenna proteins
Photosynthesis
Phenylpropanoid biosynthesis
Phenylalanine metabolism
Linoleic acid metabolism
Isoquinoline alkaloid biosynthesis
Glycolysis/Gluconeogenesis
Glutathione metabolism
Flavonoid biosynthesis
Flavone and flavonol biosynthesis
Fatty acid degradation
Biosynthesis of unsaturated fatty acids
Biosynthesis of secondary metabolites
Benzoxazinoid biosynthesis
Alpha-Linoleic acid metabolism
Alanine, aspartate and glutamate metabolism
RichFactor
Qvalue
Gene_number
DT_d0vsDT-d4
Ubiquinone and other terpenoid-quinone biosynthesis
Tyrosine metabolism
Stibenoid, diarytheptanoid and gingerol biosynthesis
Steroid biosynthesis
Starch and sucrose metabolism
Ribosome
Plant hormone signal transduction
Photosynthesis-antenna proteins
Phenylpropanoid biosynthesis
Phenylalanine metabolism
Nitrogen metabolism
Metabolic pathways
Linoleic acid metabolism
Isoflavonoid biosynthesis
Glycolysis/ Gluconeogenesis
Glutathione metabolism
Fatty acid degradation
Biosynthesis of unsaturated metabolites
Amino sugar and nucleotide sugar metabolism
Alpha-Linoleic acid metabolism
RichFactor
Gene_number
Qvalue

Supplementary Figure S4. Gene ontology enrichment of DEGs in DT and DS during time-course of drought stress

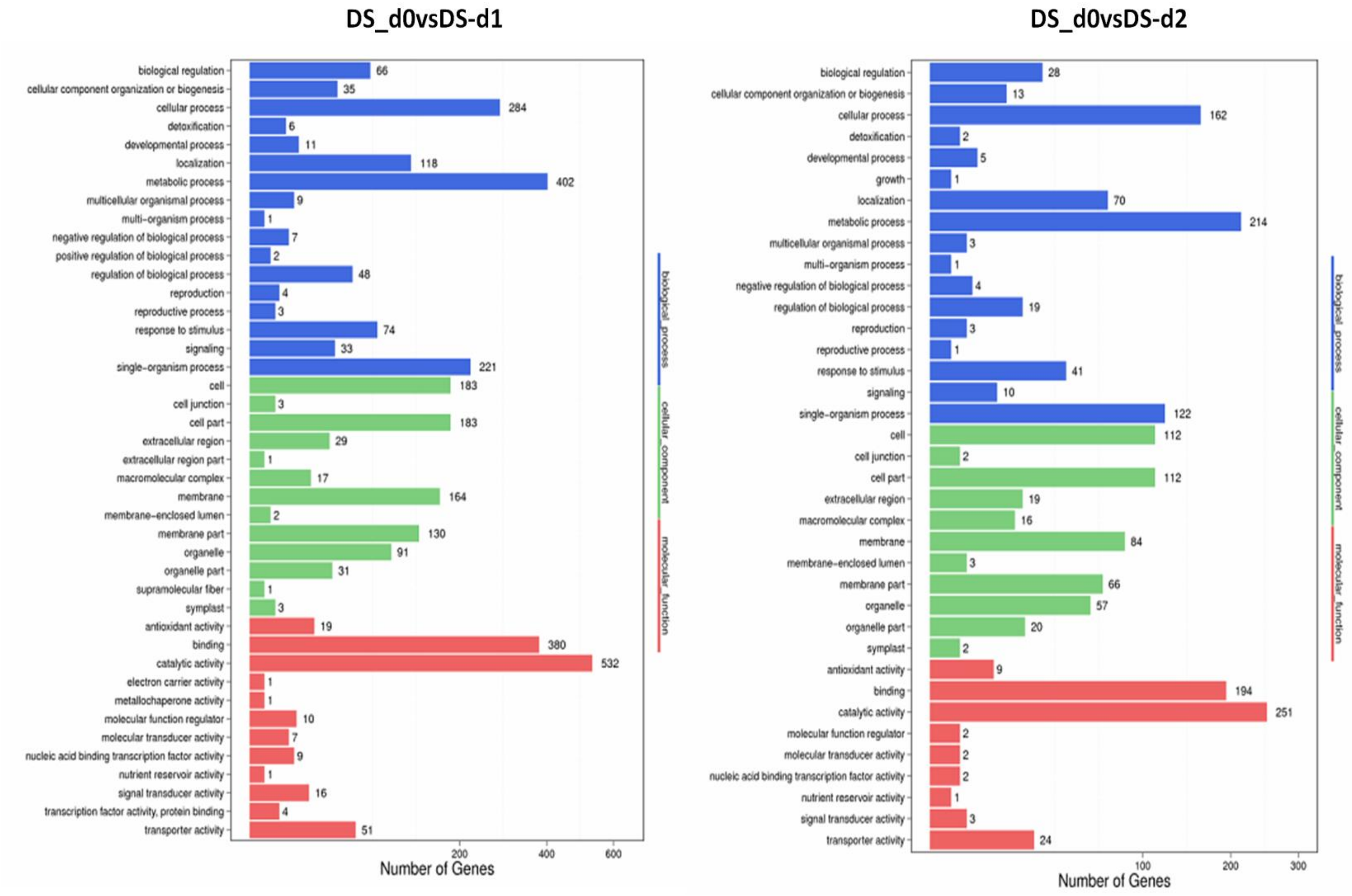

DS_d0vsDS-d3 **DT_d0vsDT-d1**

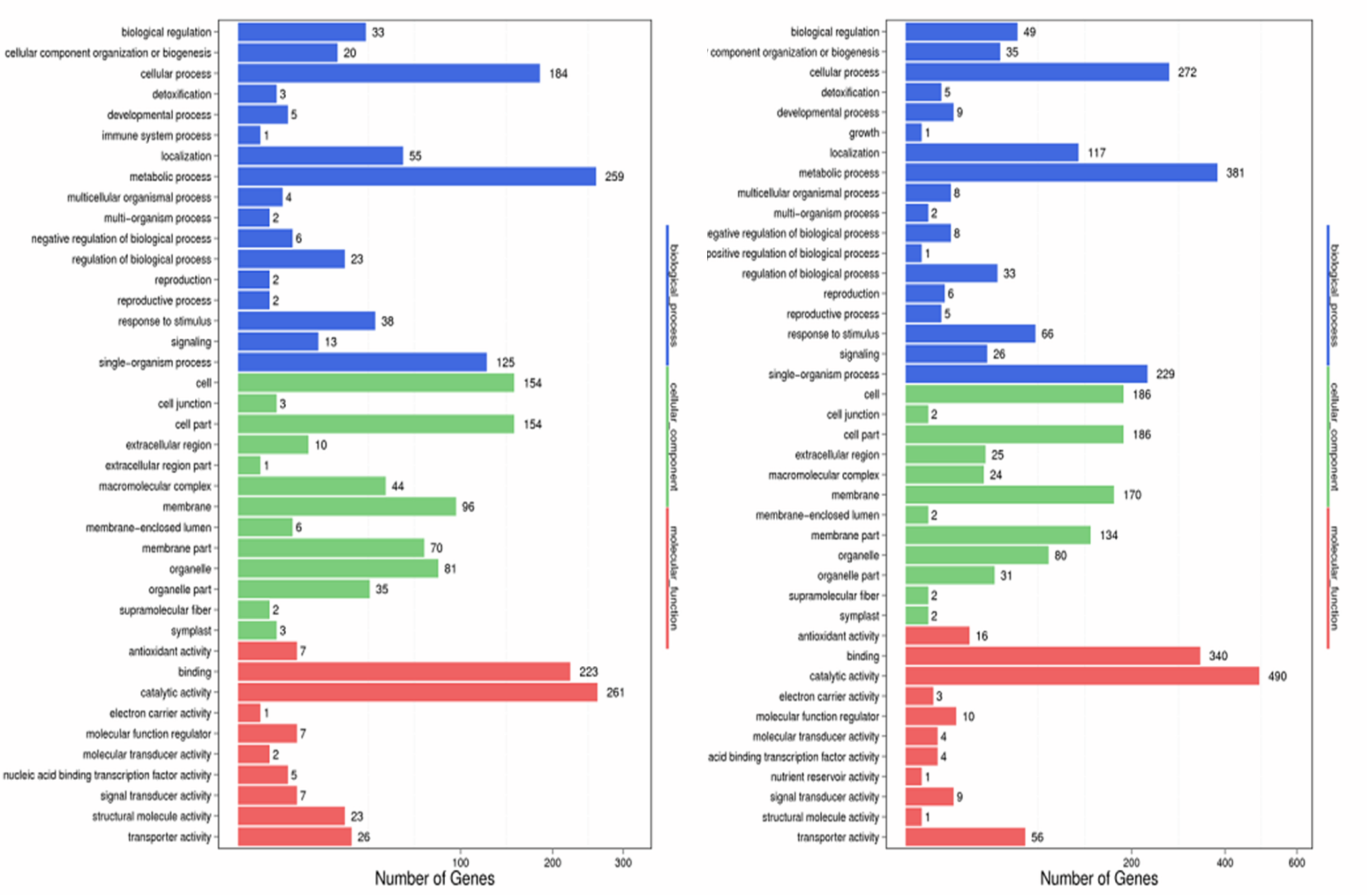

DT_d0vsDT-d2

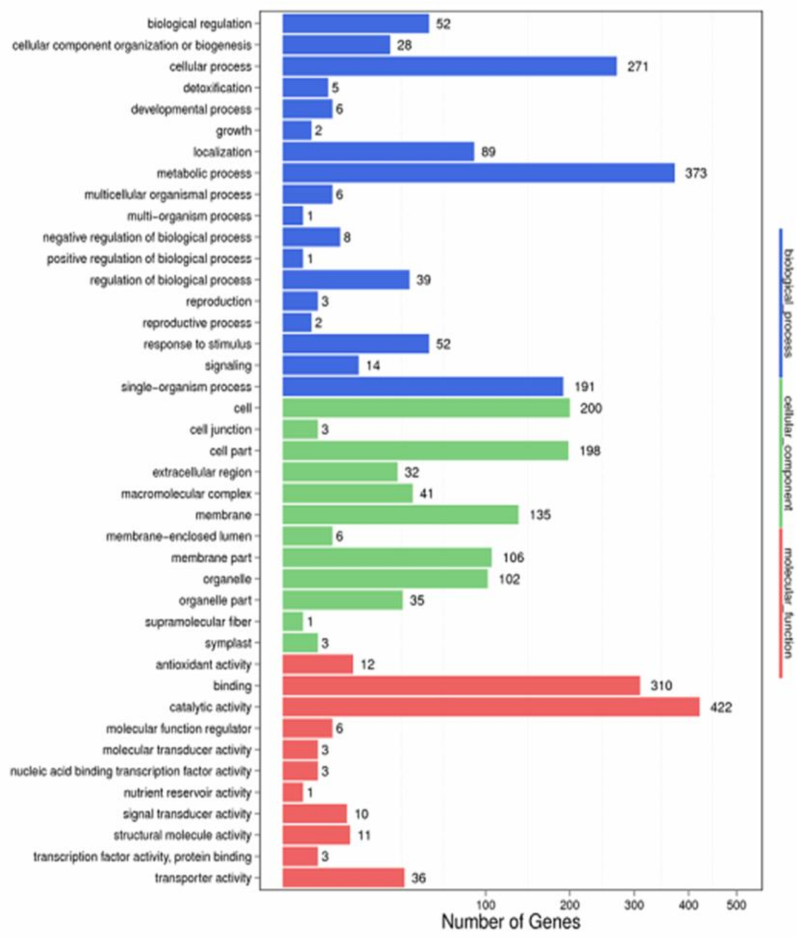

DT_d0vsDT-d3

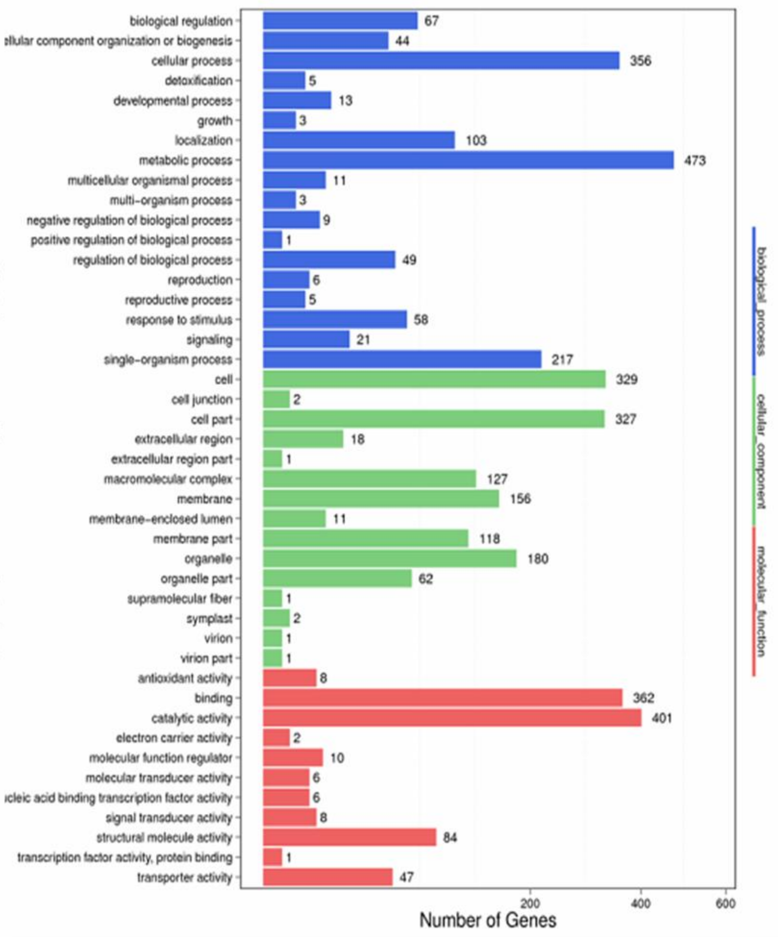

Supplementary Figure S5. Venn diagram depicting the core gene set involved in sesame response to drought

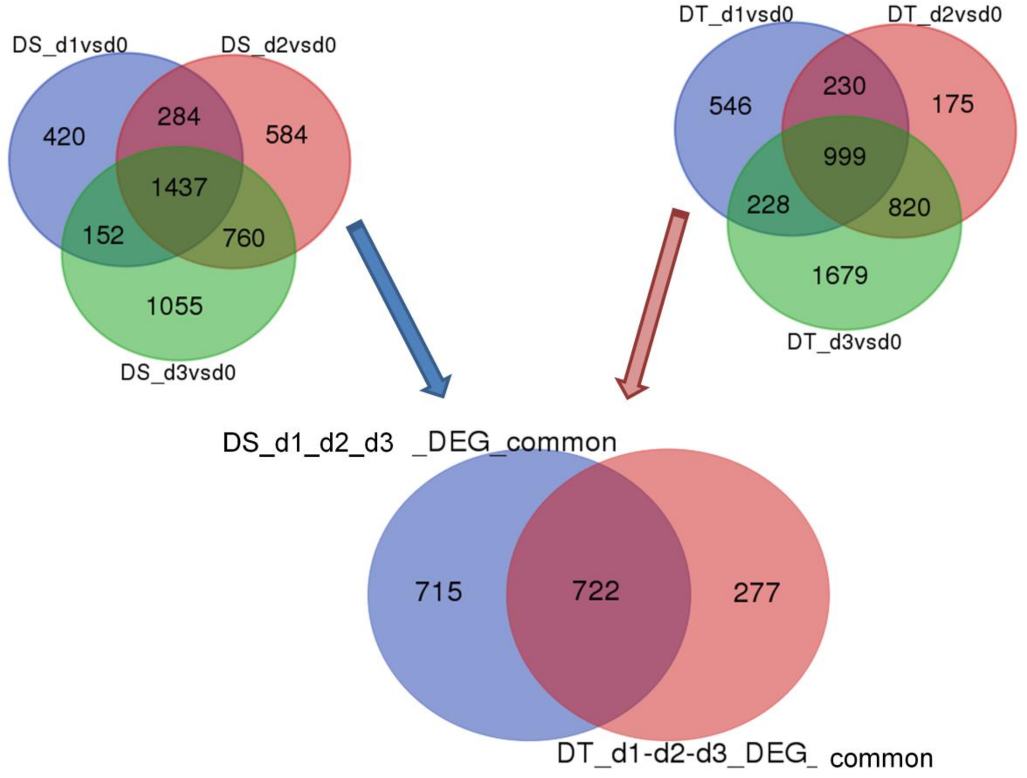

Supplementary Figure S6. Validation of selected differentially expressed genes by quantitative real-time PCR

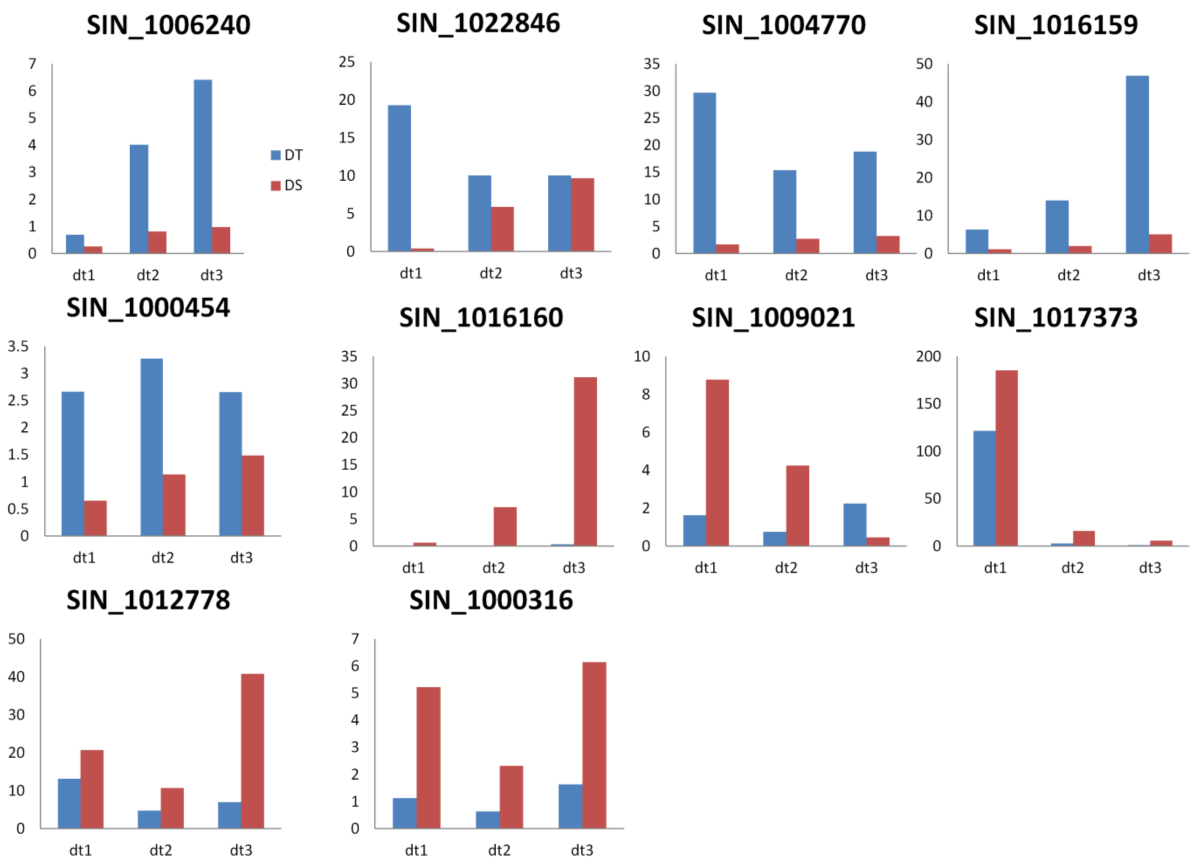

Supplement: Supplementary file 9 — Supplemenatry materials [file 41598_2017_9397_MOESM9_ESM.pdf]
